# High-Resolution pH Imaging of Living Bacterial Cells To Detect Local pH Differences

**DOI:** 10.1128/mBio.01911-16

**Published:** 2016-12-06

**Authors:** Yusuke V. Morimoto, Nobunori Kami-ike, Tomoko Miyata, Akihiro Kawamoto, Takayuki Kato, Keiichi Namba, Tohru Minamino

**Affiliations:** a Graduate School of Frontier Biosciences, Osaka University, Suita, Osaka, Japan; b Quantitative Biology Center, RIKEN, Suita, Osaka, Japan

## Abstract

Protons are utilized for various biological activities such as energy transduction and cell signaling. For construction of the bacterial flagellum, a type III export apparatus utilizes ATP and proton motive force to drive flagellar protein export, but the energy transduction mechanism remains unclear. Here, we have developed a high-resolution pH imaging system to measure local pH differences within living *Salmonella enterica* cells, especially in close proximity to the cytoplasmic membrane and the export apparatus. The local pH near the membrane was ca. 0.2 pH unit higher than the bulk cytoplasmic pH. However, the local pH near the export apparatus was ca. 0.1 pH unit lower than that near the membrane. This drop of local pH depended on the activities of both transmembrane export components and FliI ATPase. We propose that the export apparatus acts as an H^+^/protein antiporter to couple ATP hydrolysis with H^+^ flow to drive protein export.

## IMPORTANCE

The flagellar type III export apparatus is required for construction of the bacterial flagellum beyond the cellular membranes. The export apparatus consists of a transmembrane export gate and a cytoplasmic ATPase complex. The export apparatus utilizes ATP and proton motive force as the energy source for efficient and rapid protein export during flagellar assembly, but it remains unknown how. In this study, we have developed an *in vivo* pH imaging system with high spatial and pH resolutions with a pH indicator probe to measure local pH near the export apparatus. We provide direct evidence suggesting that ATP hydrolysis by the ATPase complex and the following rapid protein translocation by the export gate are both linked to efficient proton translocation through the gate.

## INTRODUCTION

Protons (H^+^) are utilized for energy and signal transduction in the complex biological networks in living cells to support various biological activities (1–3). Intracellular pH homeostasis is fundamentally essential for living cells to maintain various cellular functions. It has been reported that intracellular compartments generate a local H^+^ gradient within the cytoplasm (4) even though the diffusion coefficient of H^+^ is extremely high, estimated to be on the order of 10^−7^ to 10^−6^ cm^2^/s (5, 6). Therefore, precise measurements of local pH around biological nanomachines are critical for understanding the role of H^+^ in their biological activities.

**FIG 1:**
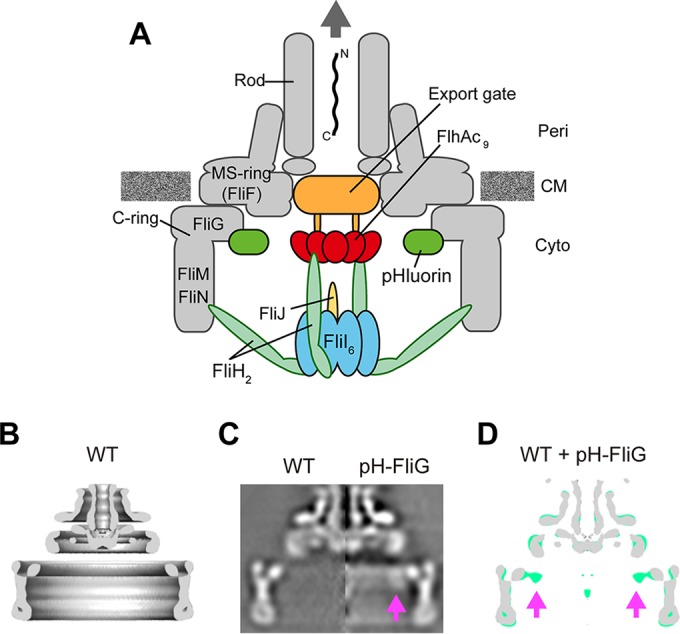
Location of the pHluorin(M153R) probe at the flagellar base. (A) Schematic diagram of the bacterial flagellar basal body with a type III export apparatus attached. The export apparatus consists of a PMF-driven transmembrane export gate made of FlhA, FlhB, FliO, FliP, FliQ, and FliR and a cytoplasmic ATPase complex consisting of FliH, FliI, and FliJ. To measure the local pH near the gate, the pHluorin(M153R) probe was fused to the N terminus of FliG. Peri, periplasm; CM, cytoplasmic membrane; Cyto, cytoplasm. (B) Averaged 3D image of the FBB purified from the *Salmonella* HK1002 strain (wild type [WT]). A c100 rotational symmetry was enforced for the refinement of the image processing. Side view of half-cut section is shown. (C) The axial sections of isolated FBB from HK1002 cells (wild type) (left) and TM041 cells [*pHluorin(M153R)-fliG*] cells (pH-FliG) (right). (D) Superposition of the wild-type FFB (gray) on the pHluorin(M153R)-FliG FBB (light green). The axial section images of the wild-type FBB and the pHluorin(M153R)-FliG FBB were processed and superimposed. The location of the pHluorin(M153R) probe is indicated by arrows. The pHluorin(M153R) position looked flexible due to the flexibility of the C-terminal region of pHluorin(M153R), thereby reducing the electron density of the pHluorin(M153R) probe.

For construction of the bacterial flagellum, which is a supramolecular motility machine, 14 different flagellar proteins are transported by a type III export apparatus to the distal end of the growing flagellar structure. The type III export apparatus utilizes ATP and proton motive force (PMF) across the cytoplasmic membrane to drive flagellar protein export (7–9). The export apparatus is composed of a PMF-driven transmembrane export gate made of FlhA, FlhB, FliO, FliP, FliQ, and FliR and a cytoplasmic ATPase complex consisting of FliH, FliI ATPase, and FliJ (Fig. 1A) (10, 11). FliH, FliI, and FliJ are not essential for flagellar protein export (12, 13), suggesting that PMF is the primary energy source. Interestingly, the export gate by itself utilizes Na^+^ as the coupling ion in addition to H^+^ when FliH and FliI are not functional. FlhA shows the H^+^ and Na^+^ channel activities, suggesting that FlhA may act as an energy transducer of the export gate, although its H^+^ channel activity is quite low (14).

The FliI ATPase forms the FliH_2_FliI complex with the FliH_2_ homodimer in the cytoplasm (15, 16). Since FliI-yellow fluorescent protein (YFP) shows rapid exchanges between the flagellar basal body (FBB) and the cytoplasmic pool in an ATP-independent manner, the FliH_2_FliI complex is proposed to act as a dynamic carrier to deliver export substrates and chaperone-substrate complexes to the export gate (17). FliI also forms the FliI_6_ ring complex at the flagellar base (18, 19). FliJ binds to the center of the FliI_6_ ring to facilitate ATP hydrolysis by FliI (20). The FliI_6_FliJ complex associates with the FBB through interactions of FliH with FlhA and a C ring component protein, FliN (21–23) (Fig. 1A). The FliH_12_FliI_6_FliJ complex is structurally similar to F- and V-type rotary ATPases, which couple ATP synthesis or hydrolysis with H^+^ translocation (16, 20, 24).

InvC is a FliI homolog of the *Salmonella enterica* virulence type III secretion system (T3SS) and has been shown to act as an unfoldase to induce the release of chaperone from the chaperone-substrate complex and to unfold the substrate for efficient protein export in an ATP-dependent manner (25). Recently, Erhardt et al. have shown that increased PMF is capable of bypassing the secretion defect of the catalytically inactive InvC(K165E) mutant strain (26), suggesting that the transmembrane export gate of the *Salmonella* T3SS has the unfolding activity operating in a PMF-dependent manner. Consistently, an increase in the cytoplasmic levels of export substrates and an increment in PMF can bypass the absence of the cytoplasmic FliH-FliI-FliJ ATPase complex considerably (26). These observations suggest that the flagellar transmembrane export gate also acts as a PMF-driven unfoldase to unfold and translocate export substrate into the 2-nm central channel of the growing structure. In agreement with this, the FliI ATPase shows no unfoldase activity (27) but ensures efficient substrate unfolding and translocation by the export gate (28). Infrequent ATP hydrolysis by FliI ATPase with the E211N substitution is sufficient for processive flagellar assembly, suggesting that ATP hydrolysis by FliI is presumably required for export gate activation (29). However, it remains unknown how.

To clarify the role of ATP hydrolysis by FliI ATPase in flagellar protein export, we developed a high-resolution pH imaging system and measured local pH near the export apparatus using a pH indicator probe, pHluorin(M153R)-FliG (Fig. 1A). We show that the local pH in the cytoplasm near the cytoplasmic membrane surface is 0.2 unit higher than the bulk cytoplasmic pH. We also show that the local pH near the export apparatus is ca. 0.1 unit lower than the pH near the cytoplasmic membrane surface and that this small drop in pH requires the presence of FliI and export gate components.

## RESULTS

### pH resolution of an *in vivo* pH imaging system.

Precise measurements of cytoplasmic pH near the export apparatus are essential for understanding the energy transduction mechanism of PMF-driven flagellar type III protein export. Therefore, we have developed a high-resolution pH imaging system that can be used for *in vivo* imaging, with a fluorescent protein, pHluorin(M153R), and a fluorescence optical microscope with a dual-wavelength illumination system. The fluorescence probe pHluorin is a ratiometric pH indicator whose emission intensities at a wavelength of 508 nm by the excitation at wavelengths of 410 and 470 nm show remarkable pH dependence over a pH range from 5.5 to 8.5, so that the emission intensity ratio (410/470 ratio) can be used to measure the pH around the probe (30). The M153R mutation was introduced to make this probe much more stable and brighter (31). The pHluorin(M153R) probe was excited by a xenon lamp equipped with a high-speed wavelength switcher that can switch the wavelength between 410 and 470 nm with a switching speed of less than 2 ms. Each fluorescent image was captured by an electron-multiplying charge-coupled device (EMCCD) camera (see Fig. S1A in the supplemental material). Our pH imaging system can measure pH over a range from 5.5 to 8.5 (see Fig. S1B). To estimate the pH resolution of our pH imaging system, the fluorescence intensities from purified pHluorin(M153R) solutions were measured at different protein concentrations and pH 7.0. The standard deviations of the observed pH values indicate that the accuracy of intracellular pH measurement is 0.02 unit (see Fig. S1C). Because the brightness of the pHluorin probe was 400 to 1,000 arbitrary units (AU) when expressed in living *Salmonella* cells, we were able to detect a pH difference in the range of 0.04 to 0.07.

### pHluorin(M153R) labeling at membrane proximity.

To carry out high-resolution pH imaging at membrane proximity, the pHluorin(M153R) probe must be localized near the cytoplasmic membrane. FliG is a C ring protein involved in flagellar motor rotation and directly associates with the cytoplasmic face of the FBB MS ring formed by 26 copies of the transmembrane flagellar protein FliF (32). The pHluorin(M153R)-FliG fusion protein is very stable and functional and is localized to the flagellar base (31). It has been shown that purified pHluorin(M153R)-FliG can measure the pH over a range from 5.5 to 8.5 (see Fig. S2A in the supplemental material). We used this fusion protein as a probe to investigate whether our pH imaging system is capable of detecting the difference between the local pH near the inner surface of the cytoplasmic membrane and the pH of the bulk cytoplasm. To identify the exact location of the pHluorin(M153R) probe in the FBB, the FBBs were purified from wild-type and *pHluorin(M153R)-fliG* strains and observed by electron cryomicroscopy (cryo-EM) (Fig. 1B). The FBB structure containing pHluorin(M153R)-FliG showed an extra density corresponding to the pHluorin(M153R) probe inside the C ring, whose diameter is 45 nm and height is 16.5 nm (Fig. 1C and D). This indicates that pHluorin(M153R)-FliG can measure not only the local cytoplasmic pH at membrane proximity but also the local pH near the export apparatus.

### *In vivo* calibration of the pHluorin(M153R)-FliG probe.

To test the capability of cytoplasmic pH measurements by the pHluorin(M153R)-FliG probe, we expressed pHluorin(M153R)-FliG in a *Salmonella* strain with deletion of the flagellar master operon *flhDC* and measured intracellular pH over an external range of 6.5 to 7.5 in the presence of 20 µM gramicidin and 20 mM potassium benzoate, by which the intracellular pH can be controlled to the same value as the external one. The pHluorin(M153R)-FliG probe was diffused in the cytoplasm (see Fig. S2B in the supplemental material), and the 410/470 ratio was the same as that of purified pHluorin(M153R)-FliG under each of the four pHs from 6.0 to 7.5 that we measured (see Fig. S2A). This indicates that pHluorin(M153R)-FliG can be used as a pH indicator probe to measure the local pH near the cytoplasmic membrane in living cells.

### Measurements of bulk cytoplasmic pH.

**FIG 2:**
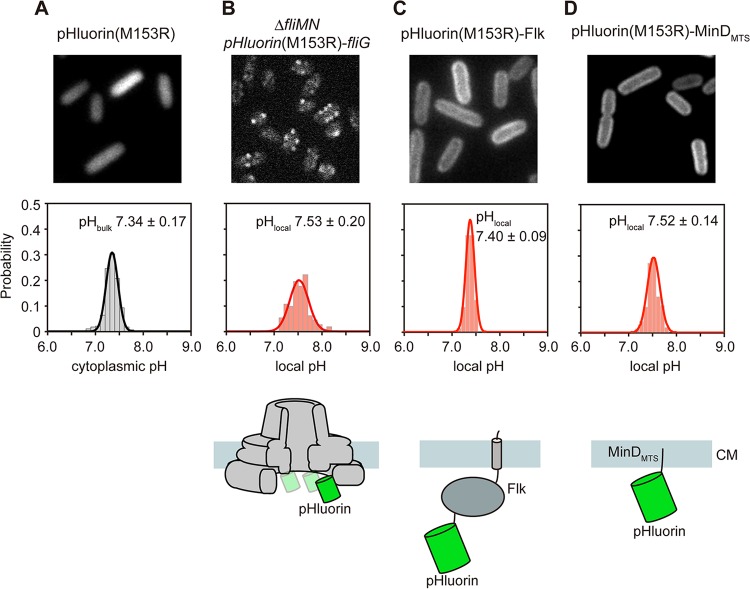
Measurements of the bulk cytoplasmic pH and local pH near the cytoplasmic membrane at external pH 7.0. (A) The bulk pH was measured with 280 wild-type cells transformed with pYVM001 [pHluorin(M153R)]. (B to D) The local pH was measured with more than 100 fluorescent spots observed with the YVMN001 cells [Δ*fliMN pHluorin(M153R)-fliG*], in which neither the complete export gate nor the MotAB proton channel complex associates with the MS ring (B), and more than 200 wild-type cells transformed with pYVM093 [pHluorin(M153R)-Flk] (C) or pYVM094 [pHluorin(M153R)-MinD_MTS_] (D). The pH distribution was fitted by a Gaussian function. Cells were incubated at 30°C in TB until the cells had reached an OD_600_ of ca. 1.6 to 1.8. Intracellular pH measurements were done at ca. 23°C. The pH values were determined by the standard curves obtained from purified pHluorin(M153R) and pHluorin(M153R)-FliG-His for bulk cytoplasmic pH and local pH, respectively.

Cytoplasmic pH is maintained at around 7.5 over a range of external pHs from 5.5 to 8.0 (33). To carry out precise measurements of the bulk cytoplasmic pH by our pH imaging system, we transformed wild-type *Salmonella* cells with a plasmid encoding pHluorin(M153R) and recorded the ratiometric pH images at an external pH of 7.0. The pHluorin(M153R) probe was diffused over the entire cell body (see Fig. S3 in the supplemental material), and the pH was measured to be 7.34 ± 0.17 (Fig. 2A; see also Table S1).

### Measurements of local cytoplasmic pH near the inner surface of the cytoplasmic membrane.

The transmembrane export gate conducts H^+^ through the FlhA proton channel to drive flagellar protein export (14, 34). The FliM-FliN complex binds to FliG to form the C ring (35–37). The MotAB complex acts as a proton channel of the flagellar motor to couple the proton influx to torque generation (38). Therefore, to precisely measure the cytoplasmic pH near the cytoplasmic membrane, the export gate, the C ring, and the MotAB proton channel complex must be removed from the FBB. FlhA assembly into the export gate depends on FliF, FliG, FliO, FliP, FliQ, and FliR (39). FlhA-YFP localizes to the FBB but not in the Δ*fliM-fliN*::*tetRA* mutant background (see Fig. S4A in the supplemental material), indicating that the Δ*fliM-fliN*::*tetRA* allele exerts a polar effect on the expression of the *fliO*, *fliP*, *fliQ,* and *fliR* genes downstream of the *fliM* and *fliN* genes. Because no MotAB complex is expressed in the Δ*fliM-fliN*::*tetRA* mutant (40), we introduced the Δ*fliM-fliN*::*tetRA* allele into the *Salmonella pHluorin(M153R)-fliG* strain by P22-mediated transduction. The pHluorin(M153R)-FliG probes formed fluorescent spots in the cells (Fig. 2B, upper panel), indicating that they bind to the cytoplasmic face of the MS ring and hence are localized close to the membrane. The local pH was measured to be 7.53 ± 0.20 in living *Salmonella* cells at an external pH of 7.0 (Fig. 2B; see also Table S1). So, the cytoplasmic pH near the membrane is about 0.2 unit higher than the bulk cytoplasmic pH (Fig. 2). To confirm this, we fused pHluorin(M153R) to a transmembrane protein (Flk) and a membrane targeting sequence (MTS) of MinD (MinD_MTS_), both of which are localized to the cytoplasmic membrane (41, 42). In agreement with previous reports, these two fusions were localized to the cytoplasmic membrane (Fig. 2C and D, upper panels). The local pH values near the membrane measured by pHluorin(M153R)-Flk and pHluorin(M153R)-MinD_MTS_ were 7.40 ± 0.09 and 7.52 ± 0.14, respectively (Fig. 2C and D, middle panels). The former value was close to the bulk cytoplasmic pH (7.34 ± 0.17) and the latter value was almost the same as that at the cytoplasmic face of the MS ring (7.53 ± 0.20). Since Flk has a relatively large cytoplasmic domain, these results indicate that the local pH values depend on a distance between the pHluorin(M153R) probe and the cytoplasmic membrane (Fig. 2, lower panels), suggesting the presence of a local pH gradient in the cytoplasm of living cells toward the membrane surface.

### Measurements of local pH near the flagellar type III export apparatus.

**FIG 3:**
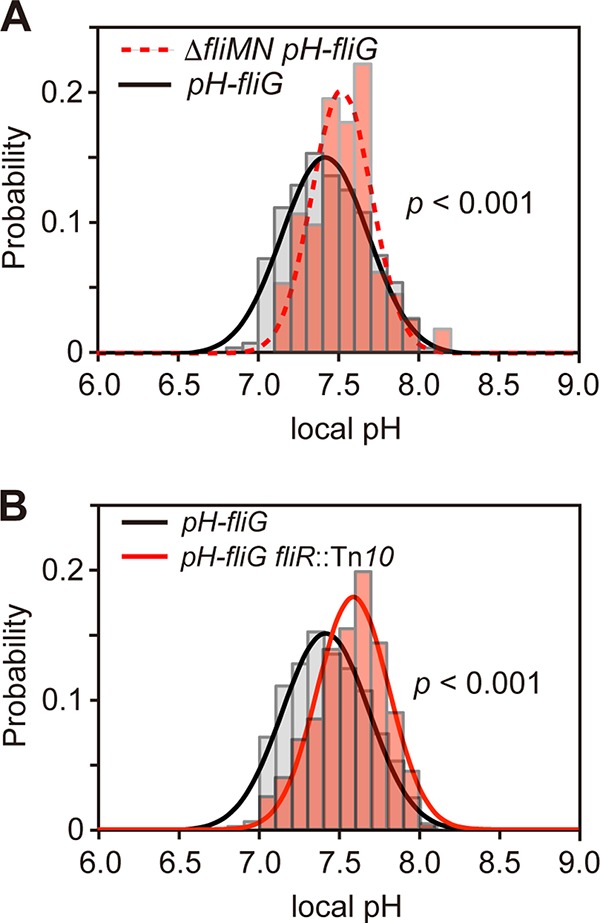
Measurements of local pH near the export apparatus. (A) Measurements of the local pH of the *pH-fliG* (YVM1004) and Δ*fliMN*::*tetRA pH-fliG* (YVMN001) cells at external pH 7.0. The local pH was measured with more than 100 fluorescent spots. The pH distribution was fitted by a Gaussian function. *P* values were calculated using a two-tailed *t* test. All cells were incubated at 30°C in TB until the cells had reached an OD_600_ of ca. 1.6 to 1.8. (B) Measurements of the local pH of the YVM1060 [*pHluorin(M153R)-fliG fliR*::Tn*10*, red line] cells at external pH 7.0. More than 200 spots were analyzed.

Next, we measured the local pH at the cytoplasmic surface of the cell membrane in the *pHluorin(M153R)-fliG* strain at external pH 7.0. The local pH near the export apparatus was measured to be 7.43 ± 0.24, ca. 0.1 unit lower than that near the membrane [Δ*fliM-fliN*::*tetRA pHluorin(M153R)-fliG* and *pHluorin(M153R)-minD*_*MTS*_] (Fig. 3A; also see Table S1 in the supplemental material). The export gate is postulated to act as an H^+^/protein antiporter (34), raising the possibility that the decrease in the local pH by 0.1 unit is a consequence of inward-directed translocation of H^+^ through the export gate. To clarify this possibility, we introduced a *fliR*::Tn*10* transposon mutation into the *pHluorin(M153R)-fliG* strain and measured the local pH near the export apparatus at external pH 7.0. Depletion of FliR did not affect the localization of FliI-YFP to the FBB (see Fig. S4B), in agreement with a previous report (22). The local pH near the export apparatus without FliR was measured to be 7.53 ± 0.24, almost the same as the local pH value measured in the Δ*fliM-fliN*::*tetRA pHluorin(M153R)-fliG* and *pHluorin(M153R)-minD*_*MTS*_ strains (Fig. 3B; see also Table S1). In contrast, in the absence of the MotAB complex, which conducts H^+^ to generate torque for flagellar motor rotation, the local pH was 7.45 ± 0.27, almost the same as the local pH near the cytoplasmic membrane measured in the *pHluorin(M153R)-fliG* strain (see Table S1), indicating that the proton channel activity of the MotAB complex does not contribute to the local pH change observed in the *pHluorin(M153R)-fliG fliR*::Tn*10* strain. The *pHluorin(M153R)-fliG* cells showed the export activity under the same experimental conditions (see Fig. S5, lane 3), indicating that the type III flagellar export apparatus is in an active state during *in vivo* pH imaging. Therefore, we suggest that a small pH drop near the functional export apparatus is due to the H^+^ influx through the export gate.

### Effects of FliI ATPase activity on local pH difference near the export apparatus.

**FIG 4:**
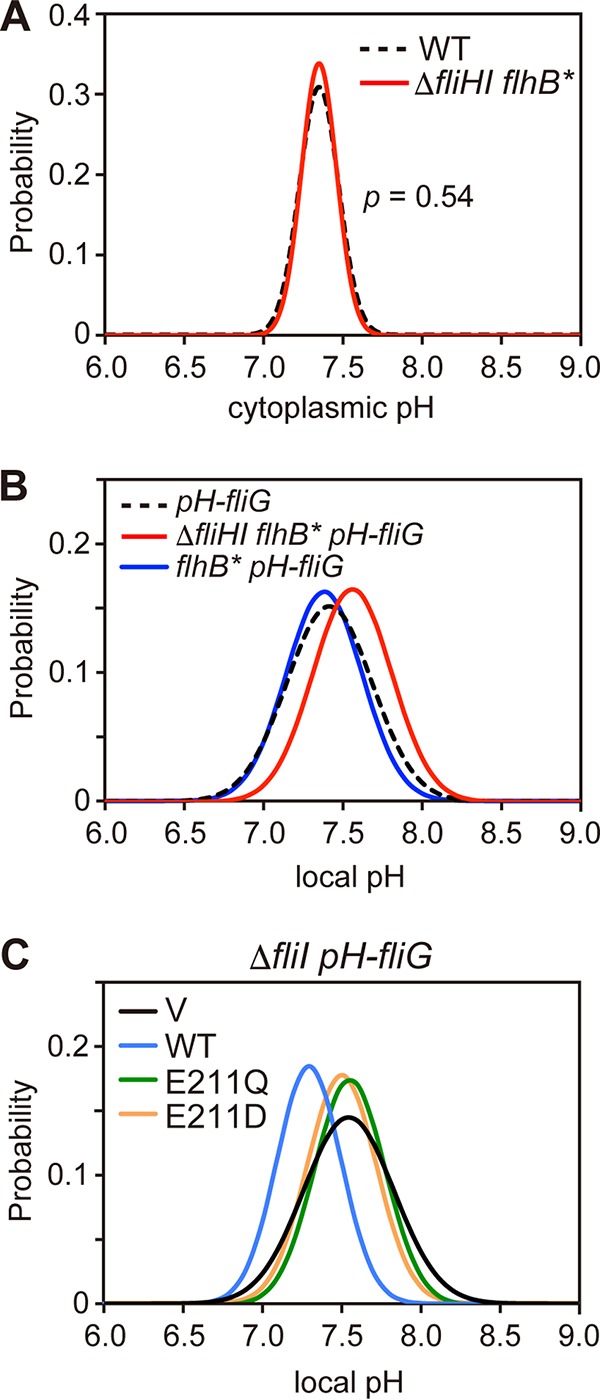
Effect of depletion of FliH and FliI on the bulk cytoplasmic pH and local pH near the export apparatus. (A) Measurements of the bulk cytoplasmic pH of SJW1103 (wild type [WT]) and MMHI0117 (ΔfliHI flhB*) cells transformed with pYVM001 at external pH 7.0 (*n* = 280 cells). *P* values were calculated using a two-tailed *t* test. (B) Measurements of the local pH of the Δ*fliH-fliI flhB(P28T) pH-fliG* (YVM1049) (red line) and YVM1063 (flhB* *pH-fliG*) (blue line) cells at external pH 7.0. The local pH was measured with more than 200 fluorescent spots. The pH distribution was fitted by a Gaussian function. The local pH distribution of YVM1004 (*pH-fliG*) is also shown by a black dashed line as a reference. All cells were incubated at 30°C in TB until the culture medium had reached an OD_600_ of ca. 1.6 to 1.8. All measurements were done at ca. 23°C. (C) Measurements of local pH of YVM1070 (Δ*fliI pH-fliG*) carrying pTrc99A (V, black line), pMM1702 (WT, light blue line), pKK211 (E211Q, green line), or pMM1702(E211D) (E211D, orange line) at external pH 7.0. More than 100 fluorescent spots were counted for each strain.

Each cell of the Δ*fliH-fliI flhB(P28T)* bypass mutant forms a couple of flagella even in the absence of FliH and FliI (12). PMF consists of the H^+^ gradient (ΔpH) and the electric potential difference (Δψ) across the cytoplasmic membrane. Only Δψ of PMF is used for flagellar protein export by wild-type cells, whereas both ΔpH and Δψ are essential for protein export by the Δ*fliH-fliI flhB(P28T)* bypass mutant (34). The ΔpH component is probably required for H^+^ movement through the export gate in the absence of FliH and FliI (34), raising the possibility that the hexameric ring complex of FliI ATPase may contribute to efficient H^+^ flow through the gate. To test this, we measured the local pH of the Δ*fliH-fliI flhB(P28T) pHluorin(M153R)-fliG* strain in motility buffer at external pH 7.0. The cells of this strain retained the export activity under the same experimental conditions (see Fig. S5, lane 4, in the supplemental material). The bulk cytoplasmic pH of the Δ*fliH-fliI flhB(P28T)* mutant was essentially the same as that of the wild type (Fig. 4A; see also Table S1). However, the local pH near the export apparatus was 0.12 unit higher in the absence of FliH and FliI [Δ*fliH-fliI flhB(P28T) pHluorin(M153R)-fliG* cells] than in their presence [*pHluorin(M153R)-fliG* cells] (Fig. 4B; see also Table S1). When FliH and FliI were expressed from the chromosomal DNA or a plasmid in the Δ*fliH-fliI flhB(P28T) pHluorin(M153R)-fliG* cells, the local pH near the export apparatus was essentially the same as that of the *pHluorin(M153R)-fliG* cells (Fig. 4B; see also Table S1). *In situ* structural analysis of the FBB of the Δ*fliH-fliI flhB(P28T)* bypass mutant by electron cryotomography and subtomogram averaging revealed that the density corresponding to the FliI ring structure was missing (see Fig. S6). Since the local pH value near the export apparatus without FliH and FliI showed a statistically significant difference compared to those near the export apparatus including FliH and FliI (*P* < 0.001) using a two-tailed *t*-test, we suggest that FliH and FliI contribute to the 0.12-unit-pH drop near the export apparatus.

To test whether the ATPase activity of FliI contributes to the drop of local pH near the export apparatus, we analyzed the effect of FliI(E211Q) and FliI(E211D) mutations on the local pH near the export apparatus. The FliI(E211Q) mutation abolishes the ATPase activity of FliI but does not affect the subcellular localization of FliI-YFP (17, 22). The FliI(E211D) mutation reduces the ATPase activity of FliI by about 100-fold (29). When FliI was expressed in the Δ*fliI pHluorin(M153R)-fliG* cells, a decrease in the local pH by about 0.13 unit was observed in comparison with the vector control (Fig. 4; see also Table S1 in the supplemental material). However, when FliI(E211Q) or FliI(E211D) was expressed, no local pH change was observed (Fig. 4C; see also Table S1). These two local pH values showed a statistically significant difference compared to that of the wild-type control (*P* < 0.001). More than 95% of the *fliI(E211Q) pHluorin(M153R)-fliG* and *fliI(E211D) pHluorin(M153R)-fliG* cells did not form flagella (see Table S1). These results indicate that both the ATPase activity of FliI and efficient flagellar formation by active flagellar protein export are required to reduce the local pH near the export apparatus by 0.1 unit compared to the local pH near the cytoplasmic membrane.

## DISCUSSION

The flagellar type III export apparatus uses ATP and PMF for efficient and rapid protein transport during flagellar assembly (12, 13). ATP is utilized only for export gate activation, allowing the gate to processively transport flagellar component proteins in a PMF-dependent manner (29, 34). Only the Δψ component of PMF is sufficient for protein export in the presence of FliH and FliI, whereas both Δψ and ΔpH become essential in their absence, indicating that these two components of PMF have distinct roles in the flagellar protein export process (34). However, it remains unknown why the export gate does not require the ΔpH component in the presence of FliH and FliI.

In this study, we showed that the local cytoplasmic pH near the membrane is about 0.2 unit higher than the bulk cytoplasmic pH (Fig. 2) and that the local pH near the export apparatus inside the C ring is 0.1 unit lower than that at the cytoplasmic surface of the membrane (Fig. 3A). We also found that such a small drop in the local pH near the export apparatus depends on both the presence of the export gate (Fig. 3B) and the wild-type ATPase activity of FliI (Fig. 4B and C). These results suggest that ATP hydrolysis by FliI and the following rapid protein translocation by the export gate are both linked to proton translocation through the gate. We did not observe the 0.1-unit drop of local pH in the Δ*fliH-fliI flhB(P28T) pHluorin(M153R)-fliG* strain (Fig. 4A), although cells of this strain retained the flagellar protein export activity under our experimental condition (see Fig. S5, lane 4, in the supplemental material). This suggests that the rate of proton translocation through the export gate would be very low in the absence of FliH and FliI. This is consistent with a recent report that the export gate prefers to utilize an Na^+^ gradient rather than an H^+^ gradient in the absence of FliH and FliI (13). Because depletion of neither the ΔpH component nor D_2_O affects the rate of protein export by wild-type cells (34), the rate of proton influx through the gate would be high enough to obscure the effect of ΔpH depletion and D_2_O in the presence of FliH and FliI. The FliH_12_FliI_6_FliJ ring complex is structurally very similar to the extra membrane part of F- and V-type ATPases (16), which hydrolyze ATP to induce an outward-directed proton pumping. Therefore, we propose that the FliH_12_FliI_6_FliJ complex and the export gate together act as an H^+^/protein antiporter to couple an inward-directed H^+^ flow through the gate with an outward-directed type III protein export and that ATP hydrolysis by FliI probably contributes to efficient inward-directed proton translocation through the gate.

FliI-YFP shows rapid turnovers between the FBB and the cytoplasmic pool during flagellar assembly (17). The FlgN-FlgK/FlgL and FliT-FliD chaperone-substrate complexes bind to FliI (43, 44). FliH and FliI ensure the interaction of an export gate protein, FlhA, with the chaperone-substrate complexes (28, 45). Since FliI enhances export efficiency under limited export substrate concentrations (26), it is also possible that an 0.1-unit pH drop around the PMF-driven export gate simply reflects the export activity of the gate powered by the H^+^ influx.

Intracellular pH homeostasis is fundamentally essential for living cells to maintain various cellular activities. Because local pH is one of the most important parameters of living cells (1–3), *in vivo* pH imaging is now becoming very important in various fields, such as medical science and pharmacology. Although fluorescent imaging of pH distribution in living cells has been done before (1, 4), we strongly believe that our *in vivo* pH imaging techniques will be a powerful tool for diverse areas of biological sciences.

## MATERIALS AND METHODS

### Bacterial strains, plasmids, and media.

*Salmonella* strains and plasmids used in this study are listed in Table S2 in the supplemental material. The *fliG* gene on the chromosome is replaced by a *pHluorin(M153R)-fliG* allele using the λ Red homologous recombination system (46) as described previously (47). L broth (LB) was prepared as described before (10). T broth (TB) contained 10 g of Bacto tryptone and 5 g of NaCl per liter. Ampicillin and arabinose were added to the medium at final concentrations of 100 µg/ml and 0.02% (wt/vol), respectively.

### DNA manipulations.

DNA manipulations were carried out as described before (47, 48). DNA sequencing reactions were carried out using BigDye v3.1 as described in the manufacturer’s instructions (Applied Biosystems), and then the reaction mixtures were analyzed by a 3130 Genetic Analyzer (Applied Biosystems).

### Flagellar protein export assay.

*Salmonella* cells were grown in 5 ml of LB with shaking at 30°C until the cell density had reached an optical density at 600 nm (OD_600_) of ~1.0. After low-speed centrifugation, the cells were washed twice with motility buffer (10 mM potassium phosphate, 0.1 mM EDTA, pH 7.0). The cell pellets were resuspended in the motility buffer and incubated at 30°C for 1 h. Cultures were centrifuged to obtain cell pellets and culture supernatants. Cell pellets were resuspended in the SDS loading buffer. Proteins in the culture supernatants were precipitated by 10% trichloroacetic acid, suspended in the Tris-SDS loading buffer, and heated at 95°C for 5 min. After SDS-PAGE, immunoblotting with polyclonal anti-FlgD and anti-FliC antibodies was performed as described before (10).

### Purification of pHluorin(M153R) and pHluorin(M153R)-FliG.

pHluorin(M153R) was purified from the soluble fractions of BL21(DE3)pLysS carrying pYVM007 as described before (33). pHluorin(M153R)-FliG-His was purified by nickel-nitrilotriacetic acid (Ni-NTA) affinity chromatography from the soluble fractions of BL21(DE3)pLysS carrying pYVM013 as described before (15, 31).

### Fluorescence microscopy.

*Salmonella* cells expressing pHluorin(M153R), pHluorin(M153R)-FliG, pHluorin(M153R)-Flk, or pHluorin(M153R)-MinD_MTS_ were observed under a custom-built microscope. An optical system was built on an inverted fluorescence microscope (IX-71; Olympus) with a 150× oil immersion objective lens (UApo150XOTIRFM; numerical aperture [NA], 1.45; Olympus) and 1.6× variable inserts and with an electron-multiplying charge-coupled device (EMCCD) camera (C9100-02; Hamamatsu Photonics). The pHluorin(M153R) probe was excited by a xenon lamp with two excitation filters, 400AF30 (Omega Optical) for 410-nm excitation and BP 470–490 (Olympus) for 470-nm excitation. A high-speed wavelength switcher (Lambda DG-4; Sutter) was used to switch between these two excitation filters with a switching speed of less than 2 ms. Fluorescence emission was passed through a dichroic mirror (FF510-Di01-25x36; Semrock) and an emission filter (520DF40; Omega Optical). Each fluorescent image was captured by the EMCCD camera. The high-speed wavelength switcher and the EMCCD camera were controlled by MetaMorph 3.6 software (Molecular Devices).

### Determination of cytoplasmic pH using pH imaging system.

Bulk cytoplasmic pH of each *Salmonella* living cell expressing pHluorin(M153R) and local cytoplasmic pH of cells expressing pHluorin(M153R)-Flk or pHluorin(M153R)-MinD_MTS_ were determined from the ratio of the fluorescence intensity in the 410-nm and 470-nm excitation wavelength images (see Fig. S7 in the supplemental material). Two fluorescence images of pHluorin(M153R) were captured by an EMCCD camera with an exposure time of 1 s for each excitation by a xenon lamp through neutral-density (ND) filters to avoid the influence of photobleaching. A set of images were analyzed with an image processing program developed based on the Igor Pro 6 (WaveMetrics) or ImageJ version 1.48 (National Institutes of Health). We defined the fluorescence intensity of the cell body determined by the image profile after subtraction of the total background intensity consisting of the instrumental background and the autofluorescence of the cell. The instrumental background intensity was defined as the mean pixel intensity of an arbitrary 100- by 100-pixel region outside the cells. The autofluorescence intensity was defined as the mean pixel intensity of 50 wild-type cells producing no fluorescent proteins. Due to an optical resolution limit by the wavelength [a peak of emission wavelength of pHluorin(M153R) is 508 nm] and numerical aperture (NA, 1.45) of the objective lens, the spatial resolution of this system is around 214 nm. Because the pixel size of the image is 33.3 nm, we carried out smoothing of each fluorescence image by processing over 7 by 7 pixels. The pH was determined using the standard curve obtained from purified pHluorin(M153R) or purified pHluorin(M153R)-FliG (see Fig. S1B). These steps were performed separately for each image.

### Determination of the local pH around the export apparatus.

Each fluorescence image was captured with a 5-s exposure. The fluorescence intensity of a single fluorescent spot of pHluorin(M153R)-FliG was determined by an integral fluorescent intensity value after subtraction of the total background intensity consisting of the instrumental background and the autofluorescence of the cell. Background threshold was determined by fitting the intensity distribution with a two-dimensional (2D) Gaussian function using a program developed on the basis of the Igor Pro 6 software (WaveMetrics) (see Fig. S8 in the supplemental material). The local pH was determined by the 410/470 ratio of each fluorescent spot. The 410/470 ratio of purified pHluorin(M153R)-FliG-His was measured by our pH imaging system at different pH values to prepare the calibration curve shown in Fig. S2A. For *in vivo* calibration, SJW1368, which is a *Salmonella flhDC* deletion strain that cannot express any flagellar genes, was transformed with pYVM008, and then, the resulting transformants were suspended in the motility buffer at various external pH values in the presence of 20 µM gramicidin and 20 mM potassium benzoate, and intracellular pH values were measured under our pH imaging system.

### Cryo-EM and image processing.

Hook-basal bodies (HBBs) with the C ring attached were prepared from two *Salmonella* strains, HK1002 and TM041, as described previously (19). A 3-μl solution of HBB was applied onto a holey carbon grid (Quantifoil R0.6/1.3; Quantifoil Micro Tools), which had been glow discharged in a weak vacuum for 5 s immediately before use. The grids were blotted twice for 3 s and quick-frozen in liquid ethane using Vitrobot (FEI). Electron cryomicroscopy (cryo-EM) images were collected as described before (19). Defocus and astigmatism in the image were determined using CTFFIND3 (49). HBB images were boxed out with BOXER (50) and aligned, classified, and averaged using the REFINE2D.PY program (49). Three-dimensional (3D) image reconstitution of the wild-type and pHluorin(M153R)-labeled HBB structures was carried out using the REFINE program with c100 symmetry (50).

### Electron cryotomography and subtomogram averaging.

Minicells of the *Salmonella* wild-type strain and the Δ*fliH-fliI flhB(P28T)* bypass mutant were prepared as described previously (19). Images of the minicells were collected at the liquid-nitrogen temperature using a Titan Krios electron microscope (FEI) operated at 300 kV and with a Falcon 4k × 4k direct electron detector (FEI) as described before (19). Images were generally binned 2-fold, and 3D reconstructions were calculated using the IMOD software package (51).

## SUPPLEMENTAL MATERIAL

Figure S1  pH imaging system using pHluorin. (A) Outline flow of pH imaging by our pH imaging system. The pHluorin probe was excited by a xenon lamp with a high-speed wavelength switcher to switch between a 410-nm excitation filter and a 470-nm excitation filter. Each fluorescent image of living *Salmonella* cells was acquired by an EMCCD camera. Intracellular pH of each cell was determined from the ratio of the fluorescence intensities of these two fluorescent cell images. (B) pH-dependent fluorescence intensity ratio of purified pHluorin(M153R). The fluorescence intensities at 508 nm of purified pHluorin(M153R) by 410-nm and 470-nm excitations were measured at different pHs under our pH imaging system. Emission intensity ratios (410/470 ratio) were plotted as a function of pH. The calibration curve was fitted by a sigmoid function. Vertical bars indicate standard deviations. (C) pH resolution. pH was determined with purified pHluorin at pH 7.0 under our pH imaging system. The standard deviations of pH were plotted as a function of the fluorescence intensity of the purified pHluorin probe with various arbitrary concentrations. The images were processed with a 7-by-7-pixel smoothing mode. A best-fit curvilinear power regression curve was selected with the Kaleida Graph 4.1 program (Synergy Software). Download Figure S1, TIF file, 0.4 MB

Figure S2  pH-dependent fluorescence intensity ratio of pHluorin(M153R)-FliG *in vitro* and *in vivo*. (A) Purified pHluorin(M153R)-His protein was observed over a pH range from 5.5 to 8.5. The 410/470 ratio was calculated at each pH value (*in vitro*). The *in vitro* data were fitted by a sigmoid function. The SJW1368 cells carrying pYVM008 were suspended in motility buffer with four distinct pH values, 6.0, 6.5, 7.0, and 7.5, in the presence of 20 µM gramicidin and 20 mM potassium benzoate, and then intracellular pH was measured under our pH imaging system (*in vivo*). Vertical bars indicate standard deviations. (B) The ratio images of the SJW1368 cells transformed with pYVM008 at external pH 6.0, 6.5, 7.0, and 7.5 in the presence of 20 µM gramicidin and 20 mM potassium benzoate. Download Figure S2, TIF file, 0.3 MB

Figure S3  Measurements of cytoplasmic bulk pH in *Salmonella* cells. (A) Cytoplasmic pH image of wild-type *Salmonella* SJW1103 cells carrying pYVM001. (B) Dependency of 410/470 ratio on the fluorescence intensity excited by 410 (left) or 470 (right) nm. Download Figure S3, TIF file, 0.4 MB

Figure S4  Subcellular localization of FlhA-YFP and FliI-YFP. (A) Effect of a *fliM-fliN* deletion on the subcellular localization of FlhA-YFP. Epifluorescence (Epi) and bright-field (BF) images of NH001 (Δ*flhA*) and YVMN003 (Δ*flhA* Δ*fliM-fliN*::*tetRA*) transformed with pYVM054 (FlhA-YFP). (B) Effect of *fliR* mutation on the subcellular localization of FliI-YFP. Epifluorescence (Epi) and bright-field (BF) images of MKM30 (Δ*fliI*) and YVMR001 (Δ*fliI fliR*::Tn*10*) transformed with pJSV203 (FliI-YFP). All observations were done at ca. 23°C. Download Figure S4, TIF file, 0.6 MB

Figure S5  Effect of pHluorin(M153R) labeling on the flagellar protein export activity of *Salmonella* cells. Immunoblotting, using polyclonal anti-FliC (upper panel) and anti-FliD (lower panel) antibodies, of whole-cell fractions (Cell) and culture supernatants (Sup) prepared from YVM1004 (*pH-fliG*) and YVM1049 (ΔfliHI flhB* pH-fliG). The positions of molecular mass markers (kilodaltons) are shown on the left. Download Figure S5, TIF file, 0.2 MB

Figure S6  *In situ* FBB structures of wild type (left) and Δ*fliH-fliI flhB(P28T)* bypass mutant (right) by electron cryotomography and subtomogram averaging. Side views of magnified views of the FBB. The density corresponding to the FliI_6_ ring is indicated by an arrow. Download Figure S6, TIF file, 0.2 MB

Figure S7  Typical imaging of the bulk cytoplasmic pH of a single cell. (A) (Upper panels) Fluorescence images of SJW1103 carrying pYVM001, excited with 410 nm and 470 nm by a xenon lamp, respectively. (Lower panels) Fluorescence intensity profiles along the straight lines indicated in the fluorescence images in the upper panels. The green areas under the peaks are used to determine the intracellular pH. (B) The 410/470 ratio of fluorescent intensities was calculated by Gaussian smoothing of 7 by 7 pixels. (C) Intracellular pH was determined from the 410/470 ratio for each pixel by the standard calibration curve measured with purified pHluorin. Download Figure S7, TIF file, 0.3 MB

Figure S8  Local pH determination with a fluorescent spot of pHluorin(M153R)-FliG. (Upper panels) Fluorescence images of YVM1004, excited with 410 nm (left panel) and 470 nm (right panel) by a xenon lamp, respectively. (Lower panels) Intensity distributions of a fluorescent spot boxed within the green square in the upper images. The integrated intensity of a single fluorescent spot of pHluorin(M153R)-FliG was calculated by fitting a 2D Gaussian function, presented as a reticular surface. Local pH was determined from the 410/470 ratio of integrated intensities of the fluorescent spot using the calibration curve (Fig. S2A). Download Figure S8, TIF file, 0.6 MB

Table S1  Measurements of local pH in various flagellar mutant backgrounds. Table S1, DOCX file, 0.1 MB

Table S2  Strains and plasmids used in this study. Table S2, DOCX file, 0.1 MB
