# Supplementary figures and images for: High-Resolution pH Imaging of Living Bacterial Cells To Detect Local pH Differences
Source: mBio. 2016 Dec 6;7(6):e01911-16. doi: 10.1128/mBio.01911-16 (PMC5142619; doi:10.1128/mBio.01911-16)

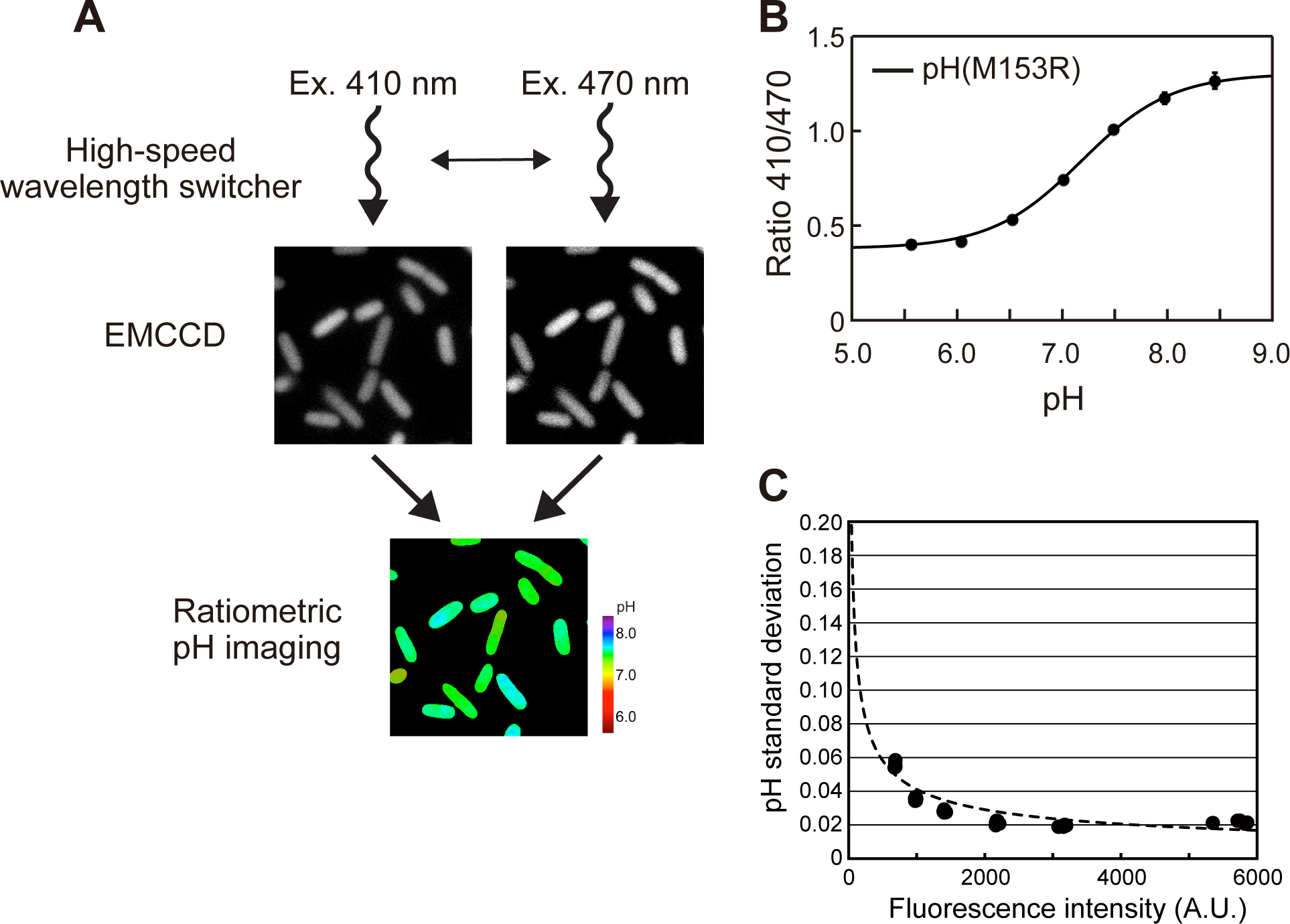

Supplement: Figure S1 — pH imaging system using pHluorin. (A) Outline flow of pH imaging by our pH imaging system. The pHluorin probe was excited by a xenon lamp with a high-speed wavelength switcher to switch between a 410-nm excitation filter and a 470-nm excitation filter. Each fluorescent image of living Salmonella cells was acquired by an EMCCD camera. Intracellular pH of each cell was determined from the ratio of the fluorescence intensities of these two fluorescent cell images. (B) pH-dependent fluorescence intensity ratio of purified pHluorin(M153R). The fluorescence intensities at 508 nm of purified pHluorin(M153R) by 410-nm and 470-nm excitations were measured at different pHs under our pH imaging system. Emission intensity ratios (410/470 ratio) were plotted as a function of pH. The calibration curve was fitted by a sigmoid function. Vertical bars indicate standard deviations. (C) pH resolution. pH was determined with purified pHluorin at pH 7.0 under our pH imaging system. The standard deviations of pH were plotted as a function of the fluorescence intensity of the purified pHluorin probe with various arbitrary concentrations. The images were processed with a 7-by-7-pixel smoothing mode. A best-fit curvilinear power regression curve was selected with the Kaleida Graph 4.1 program (Synergy Software). Download [file mbo006163091sf1.tif]

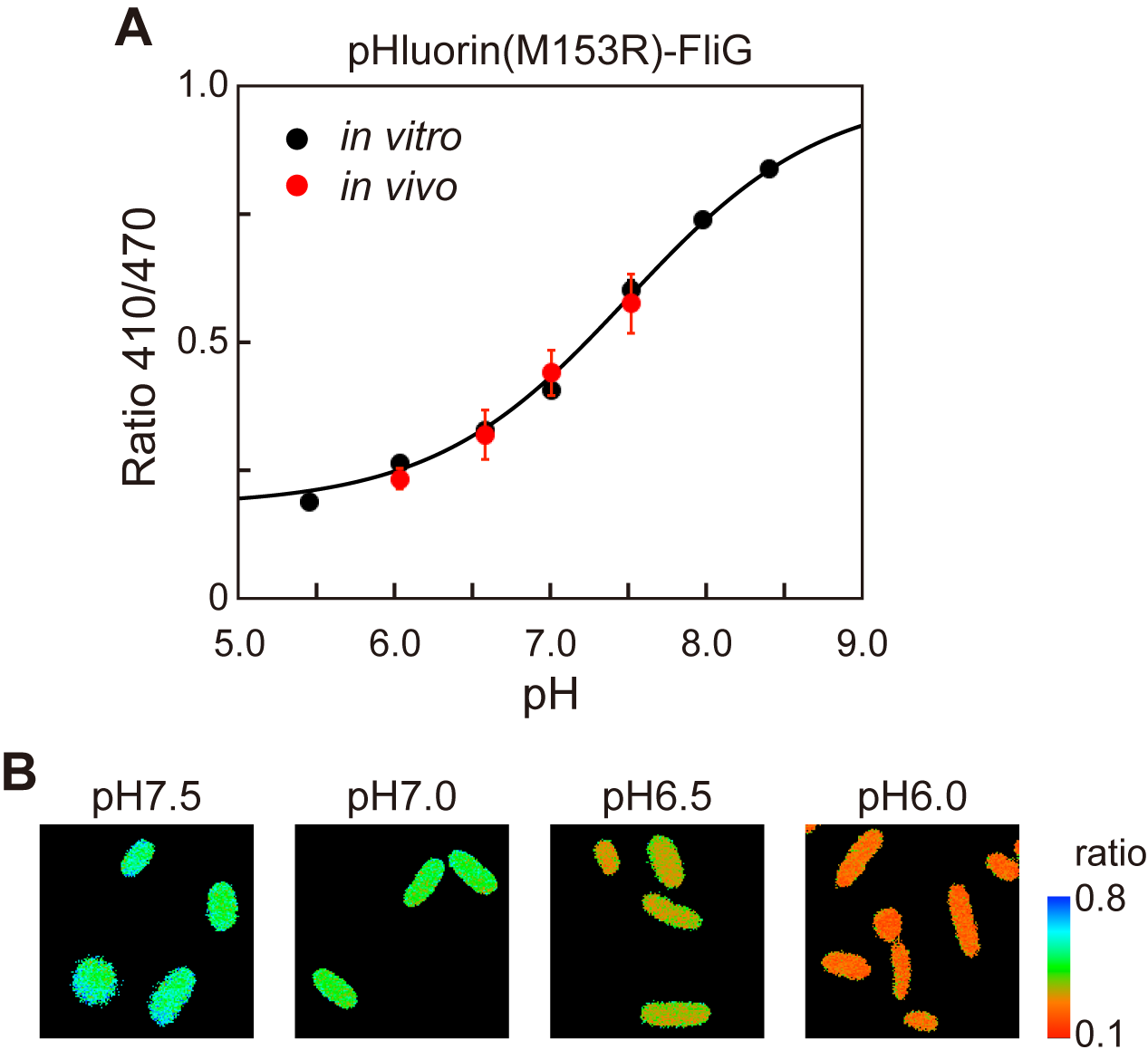

Supplement: Figure S2 — pH-dependent fluorescence intensity ratio of pHluorin(M153R)-FliG in vitro and in vivo. (A) Purified pHluorin(M153R)-His protein was observed over a pH range from 5.5 to 8.5. The 410/470 ratio was calculated at each pH value (in vitro). The in vitro data were fitted by a sigmoid function. The SJW1368 cells carrying pYVM008 were suspended in motility buffer with four distinct pH values, 6.0, 6.5, 7.0, and 7.5, in the presence of 20 µM gramicidin and 20 mM potassium benzoate, and then intracellular pH was measured under our pH imaging system (in vivo). Vertical bars indicate standard deviations. (B) The ratio images of the SJW1368 cells transformed with pYVM008 at external pH 6.0, 6.5, 7.0, and 7.5 in the presence of 20 µM gramicidin and 20 mM potassium benzoate. Download [file mbo006163091sf2.tif]

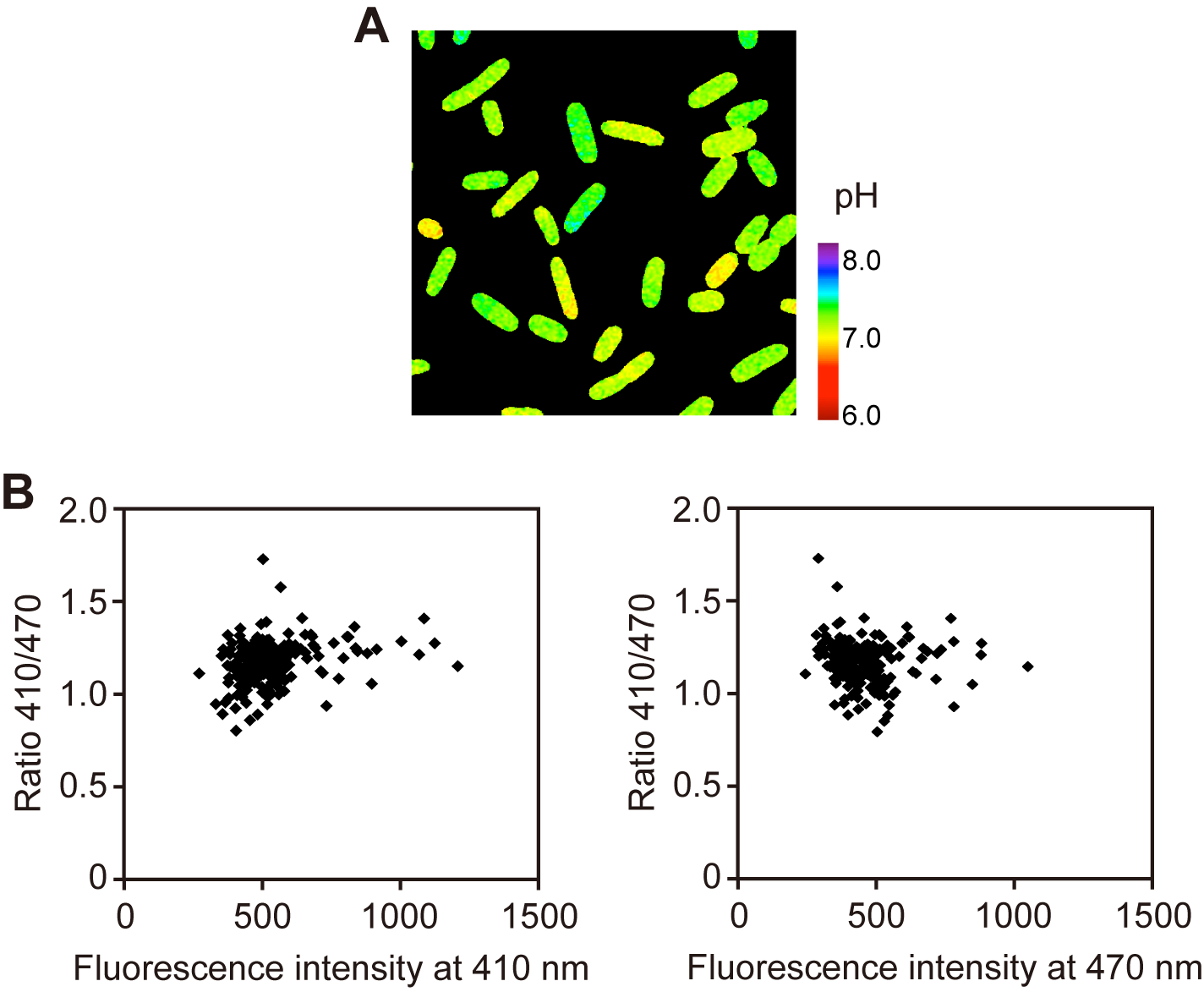

Supplement: Figure S3 — Measurements of cytoplasmic bulk pH in Salmonella cells. (A) Cytoplasmic pH image of wild-type Salmonella SJW1103 cells carrying pYVM001. (B) Dependency of 410/470 ratio on the fluorescence intensity excited by 410 (left) or 470 (right) nm. Download [file mbo006163091sf3.tif]

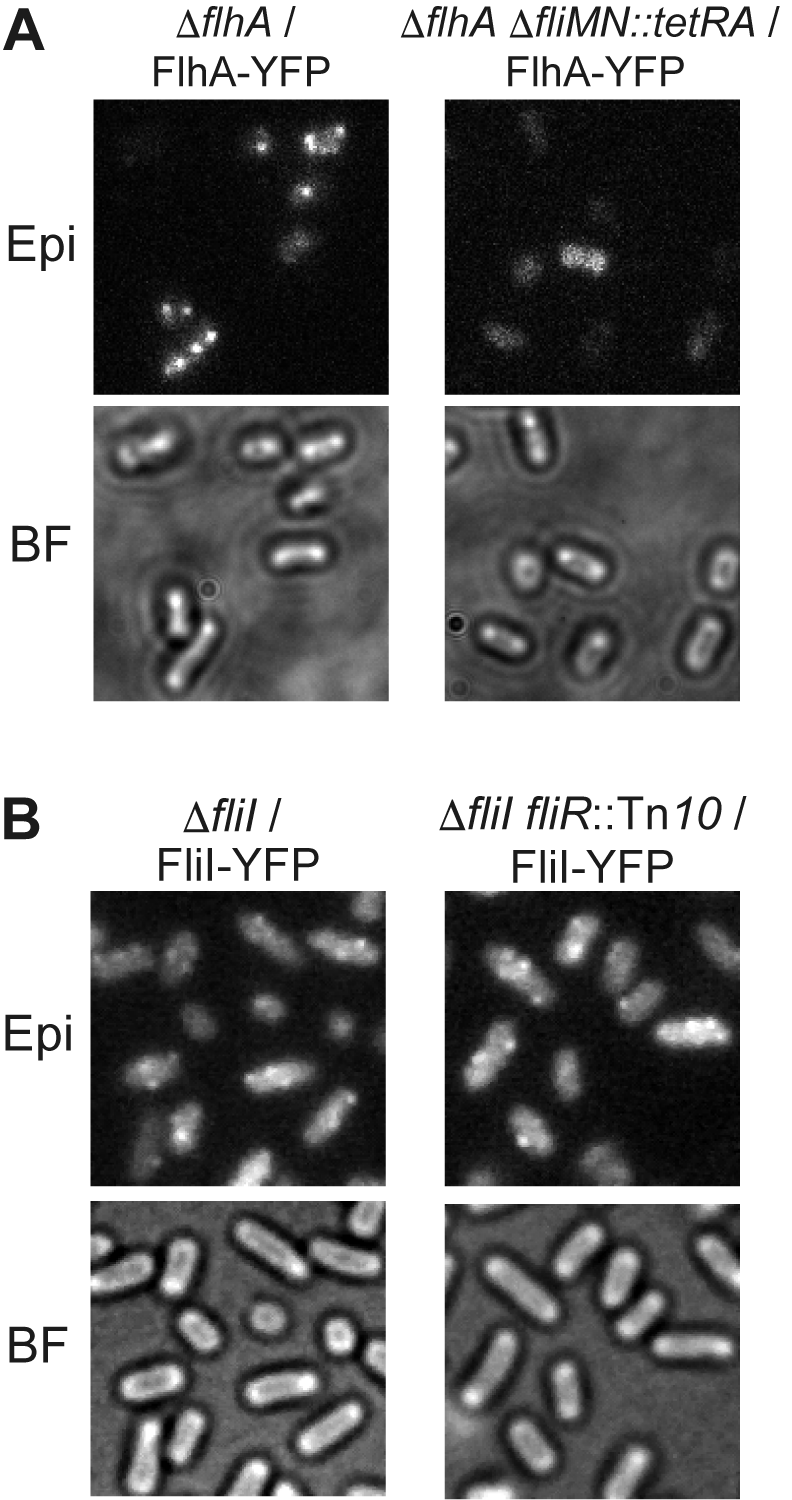

Supplement: Figure S4 — Subcellular localization of FlhA-YFP and FliI-YFP. (A) Effect of a fliM-fliN deletion on the subcellular localization of FlhA-YFP. Epifluorescence (Epi) and bright-field (BF) images of NH001 (ΔflhA) and YVMN003 (ΔflhA ΔfliM-fliN::tetRA) transformed with pYVM054 (FlhA-YFP). (B) Effect of fliR mutation on the subcellular localization of FliI-YFP. Epifluorescence (Epi) and bright-field (BF) images of MKM30 (ΔfliI) and YVMR001 (ΔfliI fliR::Tn10) transformed with pJSV203 (FliI-YFP). All observations were done at ca. 23°C. Download [file mbo006163091sf4.tif]

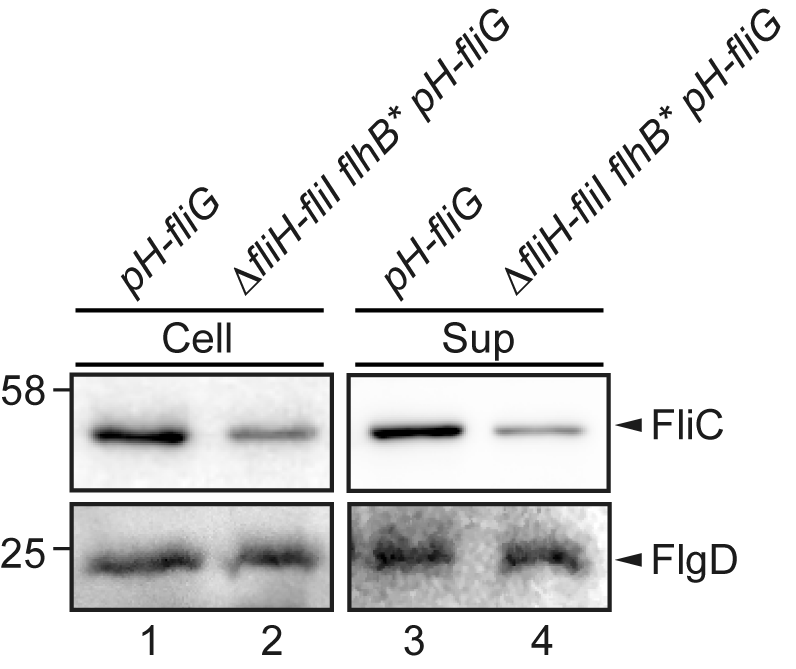

Supplement: Figure S5 — Effect of pHluorin(M153R) labeling on the flagellar protein export activity of Salmonella cells. Immunoblotting, using polyclonal anti-FliC (upper panel) and anti-FliD (lower panel) antibodies, of whole-cell fractions (Cell) and culture supernatants (Sup) prepared from YVM1004 (pH-fliG) and YVM1049 (ΔfliHI flhB* pH-fliG). The positions of molecular mass markers (kilodaltons) are shown on the left. Download [file mbo006163091sf5.tif]

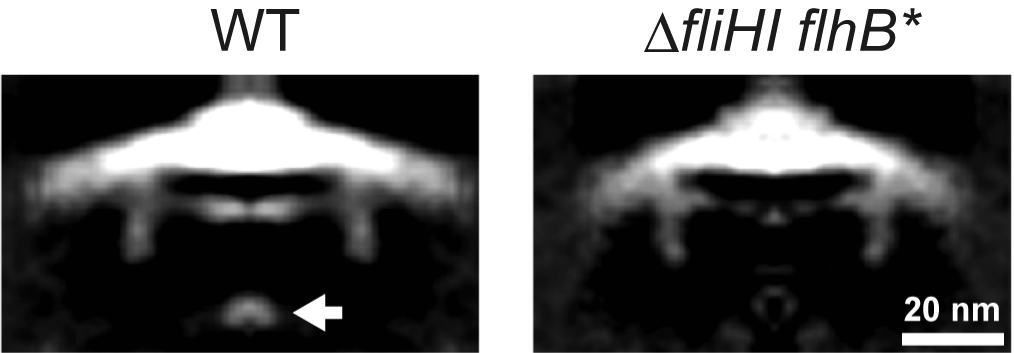

Supplement: Figure S6 — In situ FBB structures of wild type (left) and ΔfliH-fliI flhB(P28T) bypass mutant (right) by electron cryotomography and subtomogram averaging. Side views of magnified views of the FBB. The density corresponding to the FliI6 ring is indicated by an arrow. Download [file mbo006163091sf6.tif]

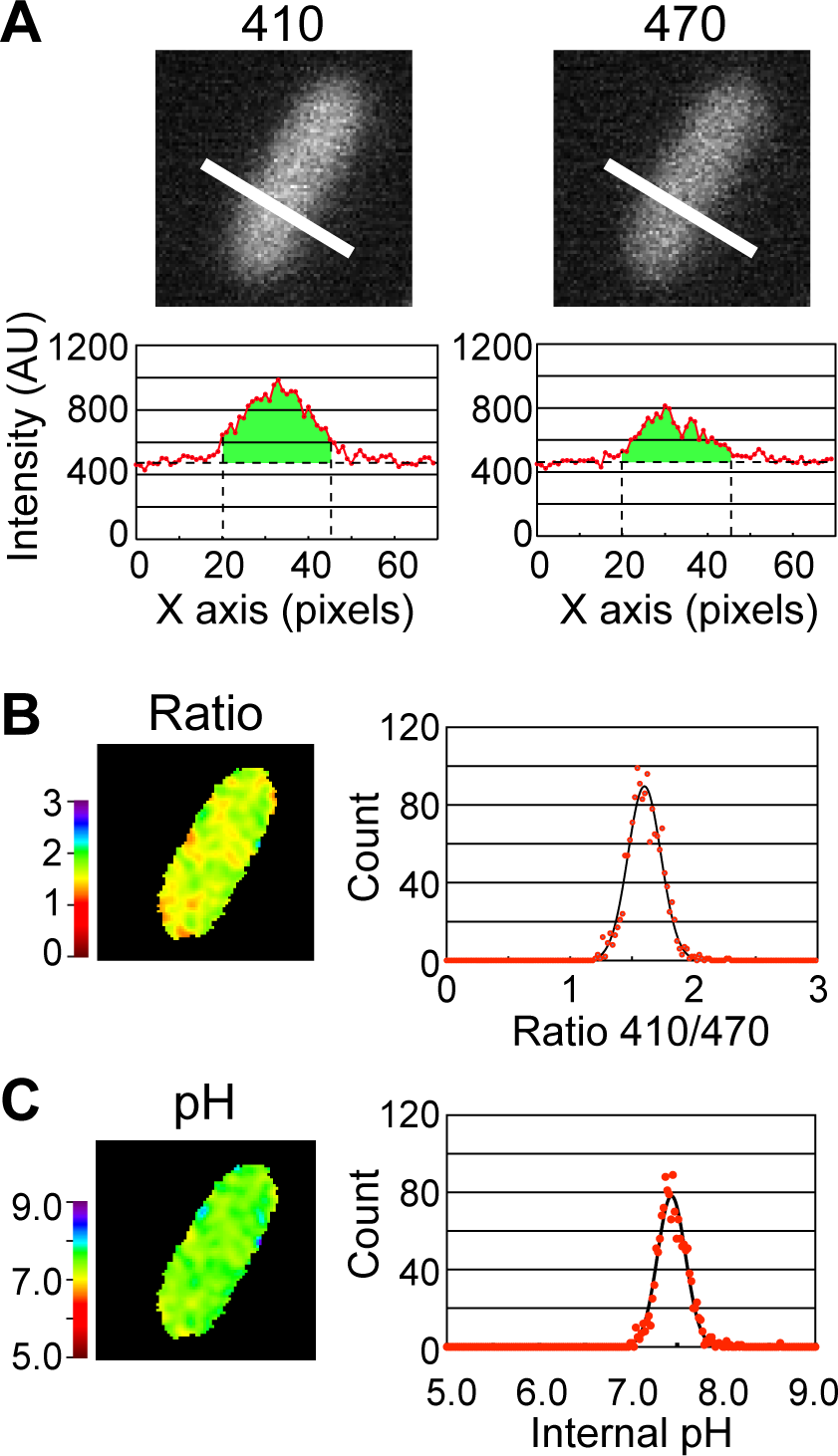

Supplement: Figure S7 — Typical imaging of the bulk cytoplasmic pH of a single cell. (A) (Upper panels) Fluorescence images of SJW1103 carrying pYVM001, excited with 410 nm and 470 nm by a xenon lamp, respectively. (Lower panels) Fluorescence intensity profiles along the straight lines indicated in the fluorescence images in the upper panels. The green areas under the peaks are used to determine the intracellular pH. (B) The 410/470 ratio of fluorescent intensities was calculated by Gaussian smoothing of 7 by 7 pixels. (C) Intracellular pH was determined from the 410/470 ratio for each pixel by the standard calibration curve measured with purified pHluorin. Download [file mbo006163091sf7.tif]

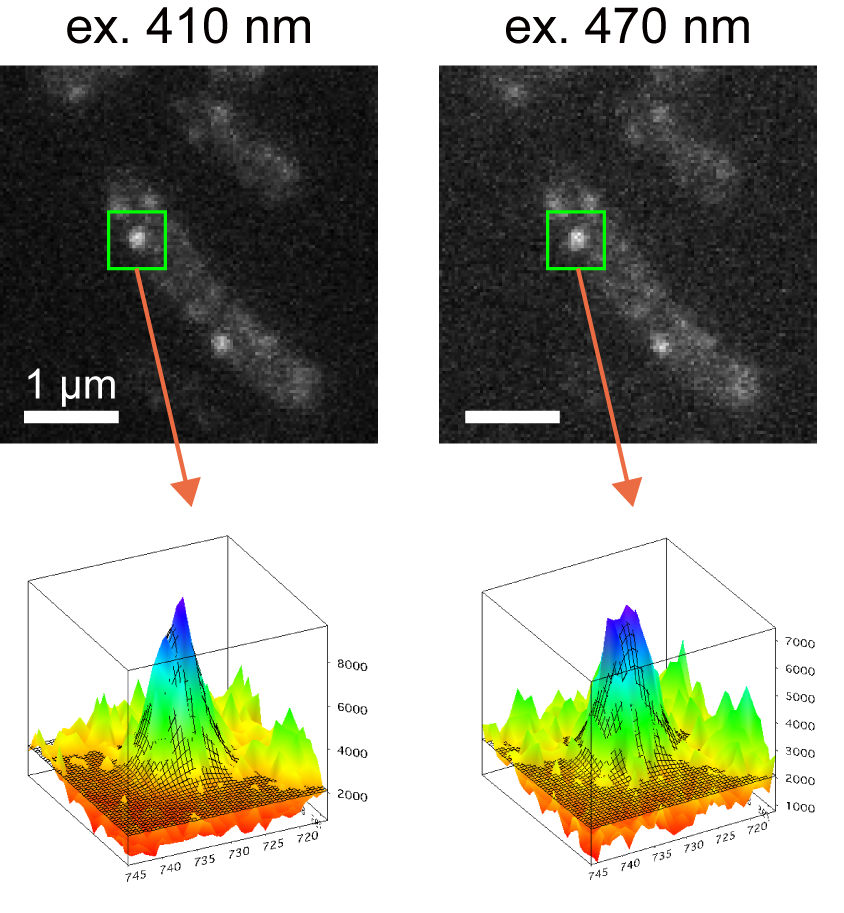

Supplement: Figure S8 — Local pH determination with a fluorescent spot of pHluorin(M153R)-FliG. (Upper panels) Fluorescence images of YVM1004, excited with 410 nm (left panel) and 470 nm (right panel) by a xenon lamp, respectively. (Lower panels) Intensity distributions of a fluorescent spot boxed within the green square in the upper images. The integrated intensity of a single fluorescent spot of pHluorin(M153R)-FliG was calculated by fitting a 2D Gaussian function, presented as a reticular surface. Local pH was determined from the 410/470 ratio of integrated intensities of the fluorescent spot using the calibration curve (Fig. S2A). Download [file mbo006163091sf8.tif]
