# Supplementary material for: High-Resolution pH Imaging of Living Bacterial Cells To Detect Local pH Differences
Source: mBio. 2016 Dec 6;7(6):e01911-16. doi: 10.1128/mBio.01911-16 (PMC5142619; doi:10.1128/mBio.01911-16)
Supplement: Table S1 — Measurements of local pH in various flagellar mutant backgrounds. [file mbo006163091st1.docx]

**Table S1** Measurements of local pH in various flagellar mutant backgrounds

|  | *Salmonella* strain characteristics | Estimated pH value | FliI ATPase activity | Export activity |
| --- | --- | --- | --- | --- |
| bulk pH | WT | 7.34 ± 0.17 | +++ | +++ |
|  | ∆*fliH-fliI flhB*(P28T) | 7.33 ± 0.15 | - | + |
|  |  |  |  |  |
| local pH | *pHluorin*(M153R)*-fliG* | 7.43 ± 0.24 | +++ | +++ |
|  | ∆*fliM-fliN::tetRA pHluorin*(M153R)*-fliG* | 7.53 ± 0.20 | +++ | - |
|  | *pHluorin*(M153R)*-fliG fliR*::Tn*10* | 7.57 ± 0.22 | +++ | - |
|  | ∆*fliH-fliI flhB*(P28T) *pHluorin*(M153R)*-fliG* | 7.55 ± 0.22 | - | + |
|  | ∆*fliH-fliI pHluorin*(M153R)*-fliG* | 7.58 ± 0.24 | - | - |
|  | *flhB*(P28T) *pHluorin*(M153R)*-fliG* | 7.42 ± 0.24 | +++ | +++ |
|  | ∆*motA-motB::tetRA pHluorin*(M153R)*-fliG* | 7.45 ± 0.27 | +++ | +++ |
|  |  |  |  |  |
| local pH | ∆*fliI pHluorin*(M153R)*-fliG*  */* V | 7.51 ± 0.23 | - | - |
|  | ∆*fliI pHluorin*(M153R)*-fliG*  */* FliI | 7.38 ± 0.23 | +++ | +++ |
|  | ∆*fliI pHluorin*(M153R)*-fliG*  */* FliI(E211Q) | 7.50 ± 0.24 | - | - |
|  | ∆*fliI pHluorin*(M153R)*-fliG*  */* FliI(E211D) | 7.52 ± 0.25 | +/- | - |
|  | ∆*fliH-fliI flhB*(P28T) *pHluorin*(M153R)*-fliG*  */* V | 7.52 ± 0.20 | - | + |
|  | ∆*fliH-fliI flhB*(P28T) *pHluorin*(M153R)*-fliG*  */* FliH + FliI | 7.41 ± 0.22 | +++ | +++ |
