# Supplementary material for: High-Resolution pH Imaging of Living Bacterial Cells To Detect Local pH Differences
Source: mBio. 2016 Dec 6;7(6):e01911-16. doi: 10.1128/mBio.01911-16 (PMC5142619; doi:10.1128/mBio.01911-16)
Supplement: Table S2 — Strains and plasmids used in this study. [file mbo006163091st2.docx]

**Table S1** Strains and plasmids used in this study

| Strain/Plasmid | Relevant characteristics | References |
| --- | --- | --- |
| *Salmonella* |  |  |
| SJW1103 | Wild type for motility and chemotaxis | (52) |
| SJW1368 | ∆(*cheW-flhD*); master operon mutant | (53) |
| MMHI0117 | ∆*fliH-fliI flhB*(P28T) | (12) |
| YVM1004 | *pHluorin*(M153R)*-fliG* | (31) |
| YVM1049 | ∆*fliH-fliI flhB*(P28T) *pHluorin*(M153R)*-fliG* | This study |
| YVM1057 | ∆*fliH-fliI pHluorin*(M153R)*-fliG* | This study |
| YVM1060 | *pHluorin*(M153R)*-fliG fliR*::Tn*10* | This study |
| YVM1063 | *flhB*(P28T) *pHluorin*(M153R)*-fliG* | This study |
| YVM1070 | ∆*fliI pHluorin*(M153R)*-fliG* | This study |
| YVM1081 | *pHluorin*(M153R)*-fliG* ∆*motA-motB*::*tetRA* | This study |
| YVMN001 | *pHluorin*(M153R)*-fliG* ∆*fliM-fliN*::*tetRA* | This study |
| YVMN003 | ∆*flhA* ∆*fliM-fliN*::*tetRA* | This study |
| YVMR001 | ∆*fliI fliR*::Tn*10* | This study |
| MKM30 | ∆*fliI* | (54) |
| YVMGK002 | *pHluorin*(M153R)*-fliG flgK*::Tn*10* | This study |
| YVMGK003 | ∆*fliH-fliI flhB*(P28T) *pHluorin*(M153R)*-fliG flgK*::Tn*10* | This study |
| HK1002 | ∆*flgK* ∆*clpP*::*Cm* | (19) |
| TM041 | *pHluorin*(M153R)*-fliG* ∆*fliC*::*tetRA* ∆*clpP*::*Cm* | This study |
| NH001 | ∆*flhA* | (47) |
|  |  |  |
| Plasmids |  |  |
| pTrc99A | Expression vector | GE Healthcare |
| pYVM001 | pKK223-3 / pHluorin(M153R) | (31) |
| pYVM007 | pGEX-2T / GST-pHluorin(M153R) | (31) |
| pYVM008 | pTrc99A / pHluorin(M153R)-FliG | This study |
| pYVM013 | pTrc99A / pHluorin(M153R)-FliG-His | (31) |
| pYVM054 | pET19b / His-FlhA-EYFP | (39) |
| pYVM093 | pBAD24 / pHluorin(M153R)-Flk | This study |
| pYVM094 | pBAD24 / pHluorin(M153R)-MinD(252–270) | This study |
| pMM1702 | pTrc99A / His-FliI | (15) |
| pMM1702(E211D) | pTrc99A / His-FliI(E211D) | (29) |
| pKK211 | pTrc99A / His-FliI(E211Q) | (55) |
| pMMHI001 | pTrc99A / FliH+FliI | (12) |
| pJSV203 | pET19b / His-FliI-YFP | (22) |

1. **Yamaguchi S, Fujita H, Sugata K, Taira T, Iino T.** 1984. Genetic analysis of H2, the structural gene for phase-2 flagellin in *Salmonella*. J Gen Microbiol **130**:255–265. doi: 10.1099/00221287-130-2-255.
2. **Ohnishi K, Ohto Y, Aizawa S, Macnab RM, Iino, T.** 1994. FlgD is a scaffolding protein needed for flagellar hook assembly in *Salmonella typhimurium*. J Bacteriol **176**:2272-2281.
3. **Minamino T, Kazetani K, Tahara A, Suzuki H, Furukawa Y, Kihara M, Namba K.** 2006. Oligomerization of the bacterial flagellar ATPase FliI is controlled by its extreme N-terminal region. J Mol Biol **360**:510–519. doi: 10.1016/j.jmb.2006.05.010.
4. **Kazetani, K. Minamino T, Miyata T, Kato T, Namba K.** 2009. ATP-induced FliI hexamerization facilitates bacterial flagellar protein export. Biochem Biophys Res Commun **388**:323–327. doi: 10.1016/j.bbrc.2009.08.004.
